# Quality Control of Fresh-Cut Apples after Coating Application

**DOI:** 10.3390/foods8060189

**Published:** 2019-06-01

**Authors:** Martina Cofelice, Francesco Lopez, Francesca Cuomo

**Affiliations:** Department of Agricultural, Environmental and Food Sciences (DiAAA) and Center for Colloid and Surface Science (CSGI), University of Molise, Via De Sanctis, I-86100 Campobasso, Italy; m.cofelice1@studenti.unimol.it (M.C.); lopez@unimol.it (F.L.)

**Keywords:** coating, surfactants, nanodispersions, essential oil

## Abstract

The growing demand for ready-to-eat fresh fruits has led to set-up appropriate strategies for preserving fruit quality and freshness of such commodities. To slow down the deterioration events such as respiration, moisture loss and enzymatic activity, ready-to-eat products should be protected with an edible film. A suitable coating should combine hydrophilic and hydrophobic features to ensure good mechanical and gas barrier properties. Alginate/essential oil nanoformulations, one with low and the other with high oil content, here proposed to protect apple pieces during storage, were first characterized through dynamic light scattering and rheology. The effect of the application of the nanoformulations on the quality parameters of apples stored at 4 °C was considered by evaluating weight loss, pH and titratable acidity, total phenols content and the fruit appearance during storage. Mainly on the basis of pH and titratable acididty variation, the nanoformulation with low oil content resulted eligible for preserving the quality of fresh-cut apple pieces during storage.

## 1. Introduction

The modification in consumers’ lifestyle and the high attention to healthy and nutritional food products enlarged the request of ready-to-eat products, such as fresh-cut fruit. The weakness of this kind of commodities is that they are perishable and difficult to preserve since minimal processing removes fruits natural protection and generates sensory deteriorations (browning, water loss, off-flavors production, loss of firmness) [1]. An appropriate strategy for ensuring food protection is the use of edible coatings, defined as primary packaging, made of edible components, such as polysaccharides, proteins, and lipids. These ingredients can be used independently or together (composite) so as to enhance the positive properties of every component. The use of complex polymer films/coatings, on the other hand, is also of central interest for other biotechnological applications [2].

Proteins and polysaccharides have excellent mechanical and structural properties and film forming ability, but they have poor water vapor and gas barrier properties. High hydrophobicity of lipids, on the other hand, provides the coating water vapor barrier features. Lipid-based edible coatings are also less permeable to gases and therefore may favor accumulation of CO_2_ and ethanol, thus favoring the development of off-flavors [3]. The positive aspects of one and the other are made available in composite systems such as emulsions. Emulsions and nanoemulsions, namely colloidal dispersions of oil droplets stabilized by a surface active agent in a continuous aqueous phase (or vice versa) [4] differ for the stability that is higher in nanoemulsions since the oil droplet diameters are typically between few tens to few hundreds of nanometers. Lately, for their interesting characteristics, nanoemulsions have been proposed as functional tools for being enriched with bioactive compounds in the lipid phase [5].

Sodium alginate is the salt of alginic acid, isolated from brown algae. It is a linear, anionic, water-soluble polysaccharide, non-toxic, biodegradable, biocompatible, food-grade, low cost and with interesting rheological properties and consists of monomeric units of 1–4 linked β-D-mannuronate and α-L-guluronate [6]. This hydrocolloid is extensively used in food formulations also as thickening or gelling agent [7].

Essential oils (EOs) are aromatic hydrophobic liquids obtained from plant parts. They can replace the use of chemical preservatives in foods while meeting the consumers demand for natural products [8,9]. The principal complication hampering their wide use is the strong impact on organoleptic properties of food. Among EOs, lemongrass essential oil (LEO) has been found to be effective against several foodborne pathogens when it was incorporated in minimally processed fruit [10,11].

The enrichment of alginate-based coatings with EOs has been recently studied [12] for applications on fish and meat products and on fresh-cut fruit. Heydari et al. in 2015 [13] studied the combined effect of sodium alginate coating and horsemint EO on the quality of refrigerated bighead carp fillets and they observed reduced spoilage of the fillets and extended shelf-life. Successively, Raeisi and coworkers [14] studied the microbial quality of chicken meat fillets during storage time by using sodium alginate active coatings incorporated with different natural antimicrobials including nisin, cinnamon and rosemary EOs, added individually and in combination. They demonstrated that all treatments significantly inhibited microbial growth when compared to the control. Along this line, Vital et al. [15] applied alginate-based edible coatings containing rosemary or oregano EOs as natural antioxidants for preserving beef steaks during 14 days. The coatings protected beef from color losses, water losses and had a positive effect on consumer acceptance. Raybaudi-Massilia et al. already in 2008, [16] incorporated malic acid and cinnamon, palmarosa and lemongrass EOs into an alginate-based edible coating to test their effects on the shelf-life of melon pieces. The coatings enriched with EOs protected the fresh-cut melon from the microbial proliferation but some fresh-cut melon characteristics were affected such as firmness and color, causing a reduction of physicochemical shelf-life. Lately, Azarakhsh and co-workers [17] investigated on how different LEO concentrations dispersed into an alginate-based edible coating influenced respiration rate, physicochemical properties, and microbiological and sensory quality of fresh-cut pineapple during 16 days of storage. The results indicated that an alginate-based edible coating formulation containing 0.3% LEO was suitable for extending the fresh-cut pineapple shelf-life. Higher concentrations of LEO, on the contrary, affected negatively the fruit quality.

The efficiency of emulsions or nanoemulsions made of alginate and LEO applied as coating on apples was studied in 2015 [18] against *Escherichia coli* proliferation during storage time. The study also focused on how the fruit respiration rate and ethylene production were influenced by LEO concentration.

Here, to investigate on how some other physicochemical quality parameters are influenced by the application of edible coatings, two nanoformulations are used for protecting fresh-cut apples. The oil in water nanodispersions were made of sodium alginate suspension (1% *w*/*w*) and LEO at two different concentrations (0.1% and 1% *w*/*w*) stabilized by the non-ionic surfactant, Tween 80. The rheological behavior of the nanodispersions was determined with rotational and oscillatory tests and the edible coating was applied on apple slices by dipping method. The effectiveness of nanodispersions as edible coating was investigated on coated fruits stored at 4 °C for 14 days while uncoated slices were used as control. Physicochemical parameters such as pH, titratable acidity, weight loss and total phenolic content were analyzed during storage.

## 2. Materials and Methods

### 2.1. Materials

Food-grade sodium alginate was obtained from Farmalabor, non-ionic surface-active agent polyoxyethylene (20) sorbitan monooleate (Tween 80), calcium chloride and citric acid were purchased from Sigma-Aldrich while Lemongrass (*Cymbopogon nardus*) Essential Oil (LEO) (Erbamea, San Giustino, Perugia, Italia) was purchased from a local shop. Fuji apples were purchased in a local supermarket. For all the preparations, ultra-pure water was used.

### 2.2. Preparation of Coating Nanoformulation

Sodium alginate (1% *w*/*w*) was dissolved in hot water at 70 °C under continuous stirring. Coarse primary dispersions were made by mixing aqueous sodium alginate suspension, LEO (0.5–5% *w*/*w*) as lipidic phase and Tween 80 (5% *w*/*w*) with a laboratory T25 digital Ultra-Turrax mixer (IKA, Staufen, Germany) with a S25N-8G probe, working at 24,000 rpm for 4 min. These dispersions were then subjected to ultrasonic treatment (Ultrasonic Homogenizer Model 300 VT, BioLogics Inc., Manassas, VA, USA) for 1 min at 120 W with 50% pulsed frequency to achieve finely dispersed hydrophobic particles providing stable suspensions [19,20]. The so obtained nanodispersions were diluted with the 1% alginate suspension in order to have a final EO concentrations of 0.1 and 1% *w*/*w* and Tween 80 concentration of 1% *w*/*w*.

### 2.3. Nanoformulations Characterization

The average size of the dispersed phase of both the nanodispersions was determined by means of dynamic light scattering (DLS) using a Malvern UK Zetasizer-Nano ZS 90 instrument operating with a 4 mW He-Ne laser (633 nm). The average diameter was measured at fixed detector angle of 90° by cumulant analysis of the autocorrelation function using software provided by the manufacturer. Before analysis samples were diluted 1:10 with ultra-pure water to avoid multiple scattering effects according to previous studies [21,22].

Rheological measurements were carried out using a rotational rheometer (Haake MARS III-Thermo Scientific, Karlsruhe, Germany) equipped with a 60 mm parallel plate geometry probe (PP60). The instrument was equipped with a Peltier element combined with a Phoenix II digital system (Thermo Scientific, Karlsruhe, Germany) for the temperature control. Samples (2.9 mL) were poured on the surface of the lower plate and the upper plate was lowered to the gap distance of 1 mm. Samples were left equilibrating for 5 min before measurements. Steady-shear flow tests were performed in controlled shear rate (CR) in the range of 0.01 to 1000 s^−1^ reached in three steps. Oscillation strain sweep measurement were carried out for determining the linear viscoelastic range (LVE) at a fixed frequency of 1Hz. Frequency sweep tests were performed using a fixed strain within the LVE and in a range of frequency from 0.1 to 100 Hz. The storage modulus (G’) and loss modulus (G”) were determinate as function of frequency. All measurements were performed at 25 °C.

### 2.4. Coating Application

**Scheme 1:**
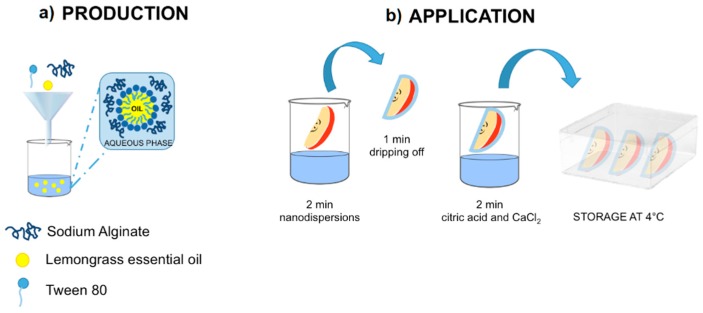
Representation of oil in water nanodispersion production (**a**) and application on apple slices surface (**b**).

Fuji apples were first sanitized by immersion in a sodium hypochlorite solution and then rinsed with tap water. 16 pieces were obtained using an apple-cutting tool. Peel and additional 2–3 mm of tissue from the core side of each piece were removed. Any small blemishes or bruises were cut away. Apple slices were dipped for 2 min into coating-forming nanoformulation and left to drip off the excess of coating dispersion for 1 min. Successively, apple pieces were dipped in a solution of citric acid 1% (*w*/*v*) and calcium chloride 2% (*w*/*v*) for 2 min as represented in Scheme 1.

Afterwards, samples were placed in polypropylene plastic trays (17 cm × 20 cm × 6.5 cm), covered with a lid, and stored at 4 °C until analyses. Uncoated apple pieces were used as control. On days 0, 3, 7, 11 and 14, three samples per treatment were taken for quality evaluation.

### 2.5. Fresh-Cut Fruit Evaluation

#### 2.5.1. Weight Loss

Apple pieces were weighed before being placed at 4 °C and at prefixed intervals (0, 3, 7, 11, 14 days). The difference between the initial weight and the weight during storage of the fruit was considered as weight loss and the results were expressed as the percentage loss of the initial weight.

#### 2.5.2. Titratable Acidity and pH

5 g of samples were taken in a beaker and homogenized with distilled water using Ultra-Turrax. The blended material was filtered and transferred in 50 mL volumetric flasks. pH of the solution was measured (pH meter). Titratable acidity (% T.A.) was determined through the titration of the apple juice with NaOH 0.1 M up to pH 8.2.% T.A. was calculated using the following formula:

(1) $$\%T.A.=\frac{V_{NaOH}\times M_{NaOH}\times E}{W\times 1000}\times 100$$

where *V* is the volume of titrant (NaOH) used, *M* is the molarity of NaOH, E is the equivalent weight of malic acid and W is the weight of sample (in grams). In case of coated samples, a thin layer was removed before the material homogenization, in order to avoid overestimation of some parameters.

#### 2.5.3. Total Phenol Content

Total phenolic content was determined according to Folin-Ciocalteu colorimetric method. 100 µL of apple juice, obtained as reported for the pH and titratable acidity, were added to 500 µL of Folin–Ciocalteu reagent and placed in the dark for 4 min. Successively, 400 µL of sodium carbonate 7.5% (*w*/*v*) were added and the cuvettes were allowed to stand in the dark at room temperature for 1 h before the detection of the absorbance at 760 nm. Caffeic acid was used as standard for calibration curve. Results are reported as ratio between the content of total phenols at certain time of storage (TPh) and their initial value (TPh_0_).

### 2.6. Statistical Analysis

SPSS software (version 23.0, IBM SPSS Statistics, Armonk, NY, USA) was used for all statistical analysis. All determinations were carried out in triplicate. Differences between treatments were tested with analysis of variance (ANOVA), followed by Tukey HSD multiple comparisons tests. Differences at *p* < 0.05 were considered significant.

## 3. Results and Discussion

### 3.1. Characterization of Coating Formulations

**Figure 1:**
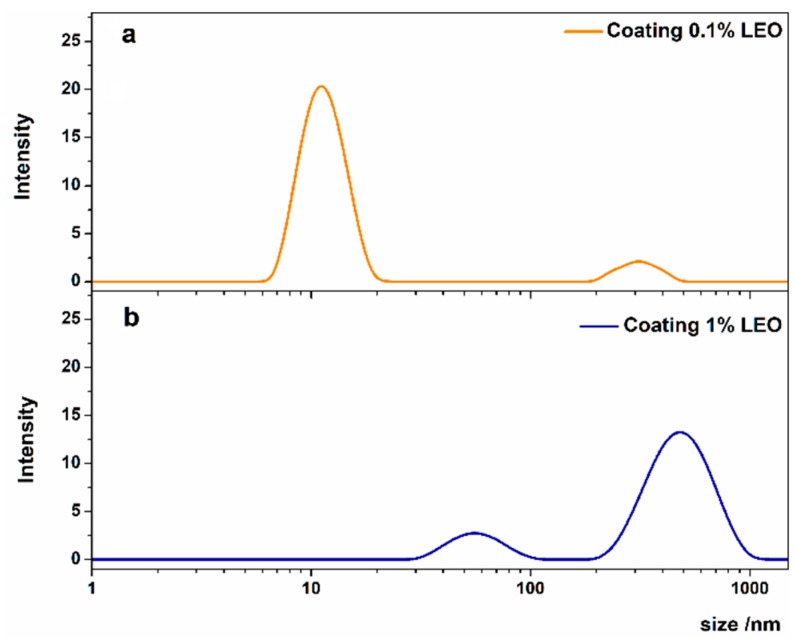
Size distributions of alginate based nanoformulations containing 0.1% LEO (**a**) or 1% LEO (**b**).

With the aim of further expanding the exploration of the applicability of LEO/alginate-based nanodispersions in routine coating experiments, we considered two type of nanodispersion that differ with respect to LEO content [12]. The two nanoformulations were characterized through DLS and rheology before coating application. From the DLS investigation (Figure 1) it was found that 0.1% LEO nanodispersions had a dispersed phase size of about 12 nm with a 0.5 value of polydispersity index (PDI). A lower homogeneity, instead, was found for the 1% LEO nanodispersion presenting two populations of aggregates around 60 and 600 nm respectively, and a higher PDI value of about 0.8.

**Figure 2:**
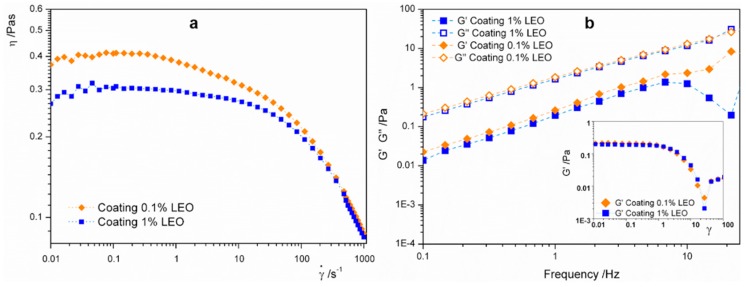
(**a**) Apparent viscosity curves as function of the shear rate for alginate based nanoformulations containing 0.1% LEO or 1% LEO; (**b**) frequency sweep spectra of nanodispersions; storage moduli *G*’ represented with full symbols and loss moduli, *G*”, represented with empty symbols. Inset shows strain sweep of nanoformulations highlighting the linear viscoelasticity region. Nanoformulation containing 0.1% LEO is represented with orange diamonds, nanoformulation with 1% LEO is represented with blue squares.

The rheological characterization of the nanodispersions was determined with rotational and oscillatory tests. In Figure 2a, flow curves of both the nanoformulations showed that apparent viscosity decreased along with the shear rate, indicating a shear-thinning behavior, generally attributable to the alignment of the polymer molecules with the flow [23]. The difference in apparent viscosity that was higher at lower LEO content can be explained with a dilution effect of alginate in the continuous phase promoted by the increase of the oil concentration [24].

Figure 2b illustrated the frequency sweep spectra carried out at a strain value extrapolated in the linear viscoelastic range (LVE) of the strain sweep (LVE range corresponds to the linear region of inset of Figure 2b). Dynamic oscillatory mechanical spectra revealed that either at high or low frequency (on short and long term) the loss modulus, *G*”, is higher than the storage modulus, *G*’, and that the moduli are highly frequency dependent. This evidence indicated that both the nanoformulations had similar liquid-like behavior compatible with the application of the coating by dipping [25]. In the further step, after the gelation of the nanoformulation promoted by calcium ions (see Scheme 1), a change to a solid-like behavior is expected [26].

### 3.2. Fresh-Cut Fruit Evaluation

**Figure 3:**
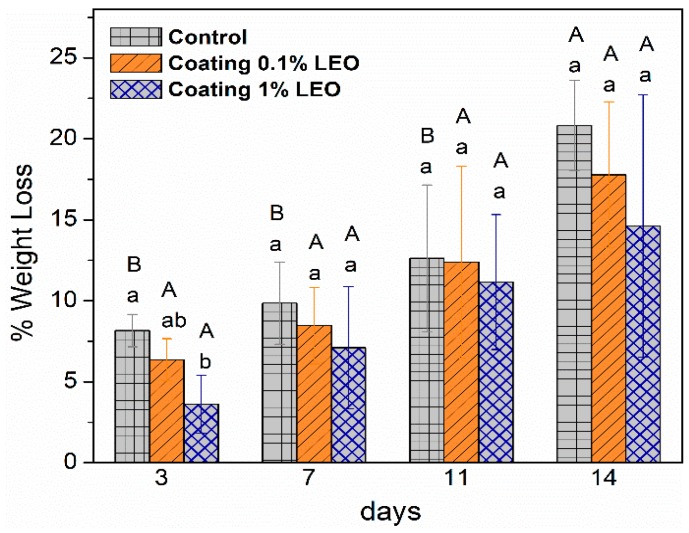
Weight loss percentage of coated and uncoated (control) fresh-cut apples with different treatment during storage at 4 °C. Means with the same letters (lowercase: amongst different treatments for the same time; uppercase: for the same treatment during different storage times) are not significantly different according to Tukey’s test (*p* > 0.05). Vertical bars indicate standard deviation.

Cutting and peeling of fruits like apples rapidly induces changes in color and appearance and over time, skinless fruit slices undergo moisture (and weight) loss. The occurrence of weight loss, an inevitable phenomenon when fruit surface is deprived of protection, is an aspect that negatively affects the quality of the product. [27,28]. Through the application of a surface coating a reduction of moisture loss, is expected. The outcomes of the weight loss evaluation are reported in Figure 3, where the moisture loss of coated apple slices is compared with uncoated pieces. As can be seen, within the first three days of storage, uncoated samples showed higher weight loss compared to coated apple pieces. The layer of coating seemed, indeed, effective in preventing water loss by producing high relative humidity at the surface of sliced apples [29]. Overall, the 1% LEO nanoformulation, in the whole storage period, reduced the weight loss more due to the higher amount of lipid phase that enhanced the barrier effect against moisture loss. Other authors [30,31,32] observed that the coating ability in reducing fruit weight loss was influenced by different permeability to water vapor of the polysaccharides used in the formulation.

The effect of coating application on pH and titratable acidity of the stored apples was also monitored. These two parameters are important to evaluate the fruit freshness and are strongly correlated because pH depends on the presence of acidic compounds. Acid content in fruits tends to decrease over time probably due to the organic acids oxidation which occurs with fruit ripening [33], as a consequence a pH increase is expected during the storage time.

**Figure 4:**
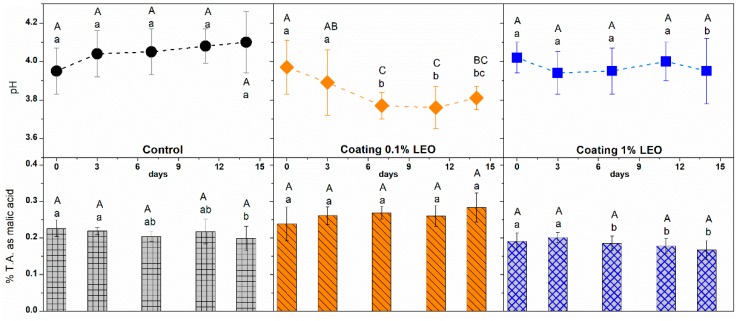
pH (upper panels) and titratable acidity (lower panels) of fresh-cut apples with different treatment during storage at 4 °C. Means with the same letters (lowercase: amongst different treatments for the same time; uppercase: for the same treatment during different storage times) are not significantly different according to Tukey’s test (*p* > 0.05). Vertical bars indicate standard deviation.

As can be observed from upper panels of Figure 4, the presence of coating reduced the pH increase and in the case of alginate-based formulations with 0.1% LEO it was even reduced in particular after the seventh day of storage. This indication can be explained considering a precise stage of the coating application corresponding to the dipping of fruit pieces in the solution containing citric acid and calcium chloride. During that step citric acid penetrated through the coating film to the apple tissues with a larger extent in 0.1% LEO than in 1% LEO nanoformulations because the presence of a higher oil content reduced, in part, the permeability to citric acid. Moreover, the evidence that fruits coated with the 0.1% LEO nanoformulation had a pH stabilized around 3.7, represents an interesting aspect because at that pH value both the polyphenol oxidase (PPO, an enzyme present in apple tissues) and the microbial activities are slowed down [34]. On the other side, titratable acidity (TA) is an important parameter that accounts for all the acids present in the fruit pieces and it is crucial to determine fruit ripening and flavor.

Lower panels of Figure 4 show the percentage of titratable acidity expressed as malic acid during storage. According to the pH evolution that has a tendency towards higher values, the TA is expected to decrease within the storage timeframe because there is an intensification of the apple respiration rate as a result of peeling, cutting and other minimal processing activities [34]. As pointed out by Soares and Fonseca [35], factors such as treatment, variety and storage conditions are all important to determinate the degree of change in acidity. Organic acids might be used as an alternative respiratory substrate during storage, leading to reduced TA levels. The latter had a specular trend compared to the pH evolution as observed by other authors [36] and apple pieces coated with the nanoformulation containing LEO at 0.1% had higher values of TA that could be associated either to the deceleration of the respiration rate or to the presence of the citric acid that contributes to preserve the fruit freshness. The higher presence of LEO in the 1% oil alginate-based formulation gave TA values almost constant within the considered time interval.

Compared to other studies, the results on pH and titratable acidity achieved with the application of the nanodispersion containing 0.1% LEO represented a positive outcome. Olivas et al. [29] that covered apple slices with alginate based coatings, enriched or not with a lipophilic phase, obtained decreasing values of titratable acidity regardless the type of coating applied. Zambrano-Zaragoza et al. [37] that applied a coating based on nanodispersion of capsules loaded with α-tocopherol also detected a decrease in titratable acidity during storage time coupled with pH increase. Salvia-Trujillo et al. [18] that used alginate-LEO emulsions and nanoemulsions as apple coating did not evaluated these parameters.

Apples, as well as many other vegetable products, represent a source of healthy compounds, such as phenols, molecules associated with antioxidant activity [38]. Chlorogenic, p-coumaric and caffeic acid, as well as quercetin, epicatechin, catechin, rutin, phlorizin are among the main phenolic compounds present in apples [39]. The quantity of phenolic compounds is highly variable, and gradients of concentration can also be found within the single fruit.

**Figure 5:**
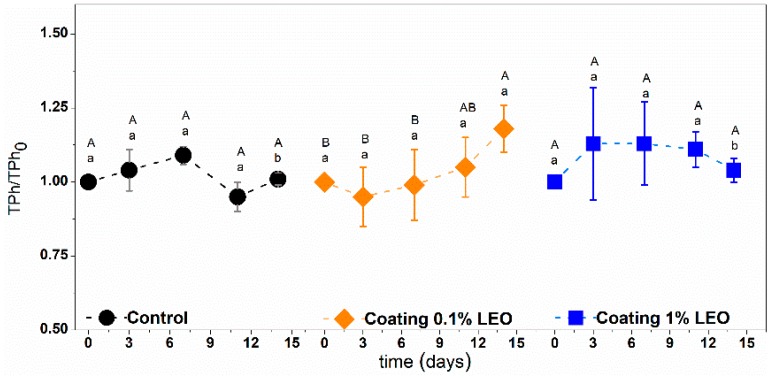
Total phenols ratio of fresh-cut apples with different treatment during storage at 4 °C. Means with the same letters (lowercase: amongst different treatments for the same time; uppercase: for the same treatment during different storage times) are not significantly different according to Tukey’s test (*p* > 0.05). Vertical bars indicate standard deviation.

Even considering the time evolution of the phenolic concentration there are no general rules for predicting accumulation or degradation because the total phenols amount depends on several aspects. An increase is, indeed, a consequence of the minimal process operations such as cutting and peeling that trigger activity of PAL (phenylalanine ammonia lyase), an enzyme that promotes the synthesis of phenols [30]. On the other hand, a decrease in phenols is due to the activity of PPO. These processes can be slowed down or inhibited by the presence of edible coatings or by the use of antioxidants. The time evolution of the phenolic compounds measured in the present study are illustrated in Figure 5 in terms of relative phenols values ratio.

The variations observed were similar even for coated apple pieces. An increase of total phenols ratio in the storage time is detected for fruit pieces covered with the 0.1% LEO nanoformulations, while at higher LEO content (1%) after an initial increase, that could be due to the essential oil migration inside the fruit tissues, constant values of total phenols were detected.

**Figure 6:**
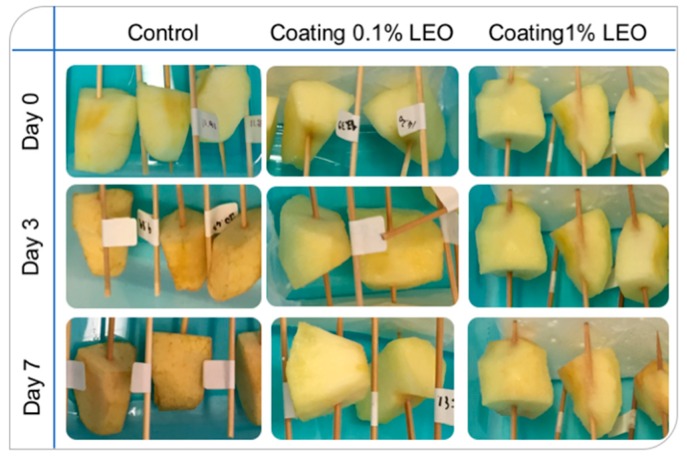
Appearance of the fresh-cut apples subjected to different treatments during storage at 4 °C.

Finally, Figure 6 illustrates the changes in fresh cut-apple pieces within 7 days of storage. As expected apple tissues underwent surface browning during storage due to the PPO activity, bringing to the formation of dark colored pigments [34].

Browning occurrence is a critical aspect that makes the produce less desirable to consumers. As can be observed from Figure 6 apple pieces coated with nanoformulations containing 0.1% LEO and 1% LEO during storage were less browned. The first row of Figure 6 shows apple pieces as soon as cut and coated, when all the samples share a good appearance. The aspect of the coated pieces was still agreeable and, in particular, apple pieces covered with 1% LEO nanoformulation seemed clearer than the others, maybe because the larger oil droplets, with their light scattering contribution added a whitening effect to the fruit pieces. During the storage period, after 3 and 7 days (second and third rows of Figure 6, respectively) the apple pieces coated with the 0.1% LEO nanoformulation were the least browned.

On the whole, as well as illustrated in Figure 6, the presence of a surface coating helps preserving the aspect of the fruit during storage. As next step, it would be useful to determine the browning index during storage according to the methodology recently assessed by other authors [40,41].

## 4. Conclusions

On balance, the present study was focused on the determination of some physicochemical fruit quality characteristics after the application of an edible coating on fresh-cut apple pieces. The coatings were realized starting from alginate-based nanoformulations containing high (1%) or low (0.1%) amount of LEO and their effects on fruit were compared with uncoated apple pieces. Both the nanoformulations had suitable rheological characteristics for being applied on fruit by dipping method, but their oil content influenced differently the fruit quality during storage. Considering the quality parameters analyzed such as the weight loss, the appearance and the positive effects detected on pH and titratable acidity, the nanoformulation with the low essential oil content (0.1% LEO) seemed more promising and appropriate than the nanoformulation with high oil content (1% LEO) for being applied on ready-to-eat fresh products.

Nevertheless, the outcomes of this study, based on the application of surface coating on fresh-cut pieces of Fuji apples, have to be considered as encouraging preliminary results to be used as starting point to further extend the application of the here proposed coating to other cultivars and to other kinds of fruit.

## References

[1] Yildiz F., Wiley R.C. (2017). Minimally Processed Refrigerated Fruits and Vegetables.

[2] Raudino M., Selvolini G., Montis C., Baglioni M., Bonini M., Berti D., Baglioni P. (2015). Polymer films removed from solid surfaces by nanostructured fluids: Microscopic mechanism and implications for the conservation of cultural heritage. ACS Appl. Mater. Interfaces.

[3] Baldwin E.A., Nisperos-Carriedo M., Shaw P.E., Burns J.K. (1995). Effect of coatings and prolonged storage conditions on fresh orange flavor volatiles, degrees Brix, and ascorbic acid levels. J. Agric. Food Chem..

[4] McClements D.J., Rao J. (2011). Food-grade nanoemulsions: Formulation, fabrication, properties, performance, biological fate, and potential toxicity. Crit. Rev. Food Sci. Nutr..

[5] Salvia-Trujillo L., Soliva-Fortuny R., Rojas-Graü M.A., McClements D.J., Martín-Belloso O. (2017). Edible nanoemulsions as carriers of active ingredients: A review. Annu. Rev. Food Sci. Technol..

[6] Cuomo F., Cofelice M., Lopez F. (2019). Rheological characterization of hydrogels from alginate-based nanodispersion. Polymers.

[7] Saha D., Bhattacharya S. (2010). Hydrocolloids as thickening and gelling agents in food: A critical review. J. Food Sci. Technol..

[8] Burt S. (2004). Essential oils: Their antibacterial properties and potential applications in foods—A review. Int. J. Food Microbiol..

[9] Rojas-Graü M.A., Soliva-Fortuny R., Martín-Belloso O. (2009). Edible coatings to incorporate active ingredients to fresh-cut fruits: A review. Trends Food Sci. Technol..

[10] Raybaudi-Massilia R.M., Rojas-Graü M.A., Mosqueda-Melgar J., Martín-Belloso O. (2008). Comparative study on essential oils incorporated into an alginate-based edible coating to assure the safety and quality of fresh-cut Fuji apples. J. Food Prot..

[11] Rojas-Graü M.A., Raybaudi-Massilia R.M., Soliva-Fortuny R.C., Avena-Bustillos R.J., McHugh T.H., Martín-Belloso O. (2007). Apple puree-alginate edible coating as carrier of antimicrobial agents to prolong shelf-life of fresh-cut apples. Postharvest Biol. Technol..

[12] Cofelice M., Lopez F., Cuomo F. (2018). Rheological Properties of Alginate–Essential Oil Nanodispersions Colloids Interfaces. Colloids Interfaces.

[13] Heydari R., Bavandi S., Javadian S.R. (2015). Effect of sodium alginate coating enriched with horsemint (*Mentha longifolia*) essential oil on the quality of bighead carp fillets during storage at 4 °C. Food Sci. Nutr..

[14] Raeisi M., Tabaraei A., Hashemi M., Behnampour N. (2016). Effect of sodium alginate coating incorporated with nisin, *Cinnamomum zeylanicum*, and rosemary essential oils on microbial quality of chicken meat and fate of *Listeria monocytogenes* during refrigeration. Int. J. Food Microbiol..

[15] Vital A.C.P., Guerrero A., de Oliveira Monteschio J., Valero M.V., Carvalho C.B., de Abreu Filho B.A., Madrona G.S., do Prado I.N. (2016). Effect of edible and active coating (with rosemary and oregano essential oils) on beef characteristics and consumer acceptability. PLoS ONE.

[16] Raybaudi-Massilia R.M., Mosqueda-Melgar J., Martín-Belloso O. (2008). Edible alginate-based coating as carrier of antimicrobials to improve shelf-life and safety of fresh-cut melon. Int. J. Food Microbiol..

[17] Azarakhsh N., Osman A., Ghazali H.M., Tan C.P., Adzahan N.M. (2014). Lemongrass essential oil incorporated into alginate-based edible coating for shelf-life extension and quality retention of fresh-cut pineapple. Postharvest Biol. Technol..

[18] Salvia-Trujillo L., Rojas-Graü M.A., Soliva-Fortuny R., Martín-Belloso O. (2015). Use of antimicrobial nanoemulsions as edible coatings: Impact on safety and quality attributes of fresh-cut Fuji apples. Postharvest Biol. Technol..

[19] Salvia-Trujillo L., Rojas-Graü A., Soliva-Fortuny R., Martín-Belloso O. (2013). Physicochemical characterization of lemongrass essential oil–alginate nanoemulsions: Effect of ultrasound processing parameters. Food Bioprocess Technol..

[20] Sánchez-Ortega I., García-Almendárez B.E., Santos-López E.M., Reyes-González L.R., Regalado C. (2016). Characterization and antimicrobial effect of starch-based edible coating suspensions. Food Hydrocoll..

[21] Guttoff M., Saberi A.H., McClements D.J. (2015). Formation of vitamin D nanoemulsion-based delivery systems by spontaneous emulsification: Factors affecting particle size and stability. Food Chem..

[22] Perugini L., Cinelli G., Cofelice M., Ceglie A., Lopez F., Cuomo F. (2018). Effect of the coexistence of sodium caseinate and Tween 20 as stabilizers of food emulsions at acidic pH. Colloids Surf. B Biointerfaces.

[23] Michel-Sanchez E. (2005). Impact of Particle Morphology on the Rheology of PCC-Based Coatings.

[24] Bonilla J., Atarés L., Vargas M., Chiralt A. (2012). Effect of essential oils and homogenization conditions on properties of chitosan-based films. Food Hydrocoll..

[25] Ferreira M.S.L., Fai A.E.C., Andrade C.T., Picciani P.H., Azero E.G., Gonçalves É.C.B.A. (2016). Edible films and coatings based on biodegradable residues applied to acerolas (*Malpighia punicifolia* L.). J. Sci. Food Agric..

[26] Liu X., Qian L., Shu T., Tong Z. (2003). Rheology characterization of sol–gel transition in aqueous alginate solutions induced by calcium cations through in situ release. Polymer.

[27] Liu X., Ren J., Zhu Y., Han W., Xuan H., Ge L. (2016). The preservation effect of ascorbic acid and calcium chloride modified chitosan coating on fresh-cut apples at room temperature. Colloids Surf. A Physicochem. Eng. Asp..

[28] Olivas G.I., Barbosa-Cánovas G.V. (2005). Edible coatings for fresh-cut fruits. Crit. Rev. Food Sci. Nutr..

[29] Olivas G.I., Mattinson D.S., Barbosa-Cánovas G.V. (2007). Alginate coatings for preservation of minimally processed ‘Gala’ apples. Postharvest Biol. Technol..

[30] Guerreiro A.C., Gago C.M.L., Faleiro M.L., Miguel M.G.C., Antunes M.D.C. (2017). The effect of edible coatings on the nutritional quality of ‘Bravo de Esmolfe’ fresh-cut apple through shelf-life. LWT Food Sci. Technol..

[31] Serrano M., Martínez-Romero D., Guillén F., Valverde J.M., Zapata P.J., Castillo S., Valero D. (2008). The addition of essential oils to MAP as a tool to maintain the overall quality of fruits. Trends Food Sci. Technol..

[32] Vargas M., Pastor C., Chiralt A., McClements D.J., González-Martínez C. (2008). Recent advances in edible coatings for fresh and minimally processed fruits. Crit. Rev. Food Sci. Nutr..

[33] Islam M., Khan M.Z.H., Sarkar M.A.R., Absar N., Sarkar S.K. (2013). Changes in acidity, TSS, and sugar content at different storage periods of the postharvest mango (*Mangifera indica* L.) influenced by Bavistin DF. Int. J. Food Sci..

[34] Rocha A., Morais A. (2003). Shelf life of minimally processed apple (cv. Jonagored) determined by colour changes. Food Control.

[35] Soares J.M., Fonseca G.G. (2008). Effect of L-ascorbic acid and sodium metabisulfite in the inhibition of the enzymatic browning of minimally processed apple. Int. J. Agric. Res..

[36] Song H.Y., Jo W.S., Song N.B., Min S.C., Song K.B. (2013). Quality change of apple slices coated with *Aloe vera* gel during storage. J. Food Sci..

[37] Zambrano-Zaragoza M.L., Mercado-Silva E., Del Real L.A., Gutiérrez-Cortez E., Cornejo-Villegas M.A., Quintanar-Guerrero D. (2014). The effect of nano-coatings with α-tocopherol and xanthan gum on shelf-life and browning index of fresh-cut “Red Delicious” apples. Innov. Food Sci. Emerg. Technol..

[38] Soares M.C., Taciana Ribeiro É., Kuskoski E.M., Valdemiro Gonzaga L., Lima A., Mancini Filho J., Fett R. (2008). Composition of phenolic acids content in apple (Malus sp) pomace. Semin. Ciências Agrárias.

[39] Heras-Ramírez M.E., Quintero-Ramos A., Camacho-Dávila A.A., Barnard J., Talamás-Abbud R., Torres-Muñoz J.V., Salas-Muñoz E. (2012). Effect of blanching and drying temperature on polyphenolic compound stability and antioxidant capacity of apple pomace. Food Bioprocess Technol..

[40] Arias E., Oria R., López-Buesa P. (2018). Determination of acceptability and shelf life of fresh-cut pear by digital image analysis. J. Food Meas. Charact..

[41] Rana S.S., Pradhan R.C., Mishra S. (2019). Image analysis to quantify the browning in fresh cut tender jackfruit slices. Food Chem..

